# A Modular PLUG‐IN Photosynthetic Chassis With Tunable Thermal Control for Mammalian Systems

**DOI:** 10.1002/advs.76102

**Published:** 2026-06-15

**Authors:** Qinhua Gan, Zheng Li, Yunfei Chen, Mingting Du, Qianyi Wu, Congcong Miao, Tao Xu, Shan Wu, Xitao Chen, Xingwei Huang, Yuhui Cheng, Chengcheng Li, Yi Xin, Yandu Lu

**Affiliations:** ^1^ Hainan Engineering & Research Center of Marine Bioactives and Bioproducts School of Marine Biology and Fisheries Haikou China; ^2^ National Key Laboratory of Tropical Crop Breeding School of Tropical Agriculture and Forestry Hainan University Haikou China; ^3^ College of Food Science of Technology Hainan University Haikou China; ^4^ Haikou Innovation Platform For Research & Utilization of Algal Bioresource Hainan University Haikou China

## Abstract

Microalgae are promising photosynthetic platforms for high‐value compounds, yet their industrial use is often hindered by a trade‐off between robust growth and the metabolic burden of payload production or cell wall disruption. Constitutive engineering for these traits compromises cultivator fitness. Here we report the development of a versatile, thermal‐regulated “PLUG‐IN” chassis in *Nannochloropsis oceanica* that enables programmable control of metabolic output and cell integrity. Comparative transcriptomics identify two highly heat‐inducible promoters (P*_NoED_* and P*_NoUK_*), which we use to construct a modular thermal gene‐amplification. Heat‐activated *AtWRI1* expression enhances triacylglycerol and eicosapentaenoic acid accumulation, while temperature‐dependent silencing of the cellulose synthase gene *CesA1* triggers rapid cell‐wall weakening without affecting growth under permissive conditions. Coupling metabolic and cell‐wall modules yields strains capable of grind‐free lipid recovery and substantially improved intracellular product accessibility. Notably, these engineered thermally controlled programs remain functional in mammalian hosts, demonstrating cross‐kingdom compatibility. This work establishes a “plug‐in” chassis compatible with mammalian systems that synchronizes growth, production, and cell‐wall re‐configuration, providing a versatile platform for photosynthetic bioproduction and microalgal synthetic biology.

## Graphical abstract

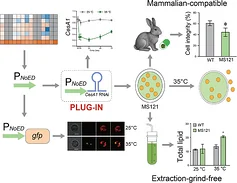

## 1 Introduction

Microalgae offer a sustainable route for biomanufacturing by directly converting light and CO_2_ into high‐value metabolites, including pharmaceutics and nutraceuticals [1, 2, 3, 4]. Among these, *Nannochloropsis* has emerged as a promising industrial chassis for producing triacylglycerols (TAGs) and omega‐3 polyunsaturated fatty acids (PUFAs), such as eicosapentaenoic acid (EPA) [5, 6, 7]. However, practical deployment of microalgal bioprocesses remains constrained by two persistent challenges: (i) the inability to independently regulate growth and production phases, and (ii) the presence of rigid cell walls that severely limit product recovery and bioaccessibility [8].

Traditional strategies to induce lipid overaccumulation (nutrient deprivation, high light, or heat shock) inevitably impair growth, enforcing a trade‐off that restricts productivity [5, 9, 10, 11]. Likewise, mechanical or chemical cell disruption required for product extraction is energy‐intensive, costly, and incompatible with sensitive biomolecules [12, 13, 14, 15]. These obstacles highlight the need for rewritable, condition‐dependent genetic programs that allow microalgal cells to grow robustly yet activate production and self‐release of intracellular metabolites on demand [1].

Temperature is an attractive regulatory input for industrial cultivation due to its scalability, precision, and compatibility with existing algal cultivation infrastructures [16, 17]. While thermal‐responsive gene switches have been developed in yeast, plants and mammalian systems, analogous regulatory tools remain largely unexplored in microalgae [18, 19, 20]. Harnessing endogenous heat‐responsive promoters could provide a powerful mechanism to temporally coordinate biomass accumulation, metabolic induction and controlled cell disassembly.

Here, we report a temperature‐responsive “plug‐in” chassis in the promising synthetic biology host, *N. oceanica* [21, 22, 23, 24]. By leveraging comparative transcriptomics, we identify two strong heat‐inducible promoters, P*_NoED_* and P*_NoUK_*, and engineer them to independently control lipid biosynthesis and cell‐wall remodeling modules. Thermal activation of the transcription factor *AtWRI1* significantly boosts TAG and EPA accumulation, while temperature‐dependent silencing of *CesA1* encoding cellulose synthase enables inducible fragilization of algal cell walls without compromising growth. Integrating these modules yields strains capable of high‐yield lipid production and grind‐free extraction. Surprisingly, the thermal‐controlled program is also activated within mammalian gastrointestinal environments, demonstrating robust cross‐kingdom functionality. Together, this work introduces a thermally regulatable system that functions as a programmable “plug‐in” unit for photosynthetic chassis engineering. As a proof of concept, the system provides a generalizable strategy for linking growth, metabolic induction, and host accessibility, enabling new opportunities in microalgal biomanufacturing, sustainable production of value‐added compounds, and living delivery platforms.

## 2 Results

### 2.1 Identification of Thermal‐Inducible Promoters in N. Oceanica

Because the core body temperature of most mammals ranges from 35°C to 38°C [25, 26], 35°C represents a biologically relevant and practically deployable threshold for thermally controlled gene expression. The growth *N. oceanica* is dramatically inhibited at 35°C and almost abolished after two days (OD_750_ and cell density, Figure S1a,b). Moreover, a sharp decline of the *F*v/*F*m values (the maximum quantum yield of photosystem II, which is an indicator of stress tolerance) was observed after the transfer of the microalgae from 25°C to 35°C (Figure S1c). Therefore, 35°C was employed as a “thermally inducible gene switch” for the microalgae.

We then analyzed time‐series transcriptomes of *N. oceanica* exposed to 25°C and 35°C at 0, 3, 24 and 48 h to identify promoters responsive to this physiologically meaningful temperature shift. A total of 4046 genes (∼40% of the genome) displayed significant temperature‐dependent expression changes (≥2‐fold up‐regulation or \\≤0.5‐fold down‐regulation). Although the early transcriptome response was modest (1490 genes at 3 h; 1150 at 24 h), a broad and orchestrated genome‐wide response emerged at 48 h (3144 genes), indicative of a robust acclimation program (Figure S2a). Functional categorization grouped differentially expressed genes into 31 subcategories across four major clusters—signaling, metabolism, genetics, and environmental responses (Figure S2b,c). Up‐regulated genes were strongly enriched in transcriptional and translational pathways (Figure S2b), whereas down‐regulated genes primarily involved metabolic and genetic modules (Figure S2c). These findings suggest that the promoters of late‐induced genes represent strong candidates for constructing thermally programmable expression systems.

**FIGURE 1:**
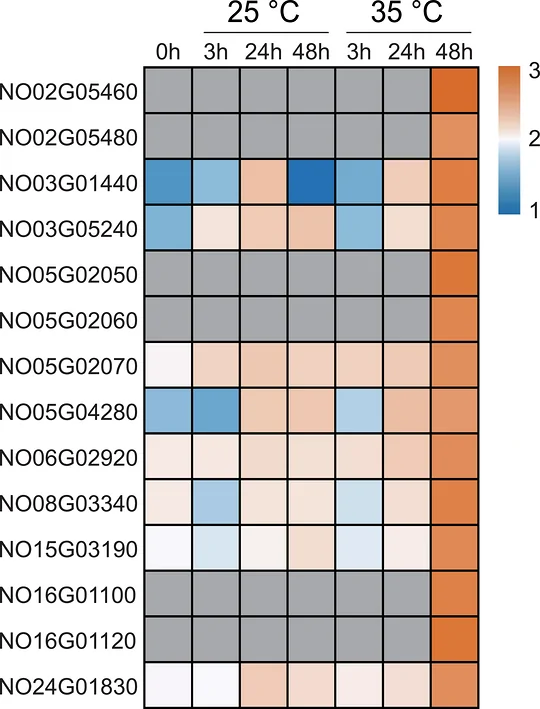
Identification of key genes involved in the heat‐stress response. Transcriptomic responses of *Nannochloropsis oceanica* cultured at 25°C or 35°C were profiled at 0, 3, 24, and 48 h. Fold changes were calculated as log_10_FPKM (Tx), where FPKM denotes normalized transcript abundance at each time point (Tx). Grey blocks indicate an FPKM value of zero. *Note*: NO02G05460, NO02G05480, NO08G03340, NO15G03190, and NO24G01830 encode proteins of unknown function (NoUKs); NO05G02050, NO05G02060, and NO05G02070 encode components of the cytochrome *b*_6_ *f* complex; NO16G01100 and NO16G01120 belong to the late embryogenesis abundant protein family; NO03G01440 encodes a diacylglycerol acyltransferase; NO03G05240, NO05G04280, and NO03G01440 encode a zinc‐finger protein, a NAD‐dependent epimerase/dehydratase (NoED), and a heat‐shock protein (HSP100), respectively.

To identify the most stringent heat‐inducible promoters, the 3144 heat‐responsive genes (Figure S2a, right) were filtered based on strict expression criteria: low basal expression (FKPM < 20 at 0 h), minimal expression at 25°C (FKPM < 50 at 48 h), and strong induction at 35°C (FPKM > 3000 at 48 h). This yielded 14 candidate promoters (Figure 1), representing genes encoding putative unknown proteins, cytochrome b_6_f complex subunits, LEA family proteins, a zinc‐finger protein, a NAD‐dependent epimerase/dehydratase (NoED), a diacylglycerol acyltransferase (NoDGAT2F), and one heat‐shock protein (HSP100). Transcriptomic activation is modest at early time points (3 h) and becomes pronounced during sustained exposure. Collectively, these candidates provided a diverse regulatory palette for designing thermal‐activated modules.

### 2.2 Validation of Heat‐Inducible Promoters In Vivo

**FIGURE 2:**
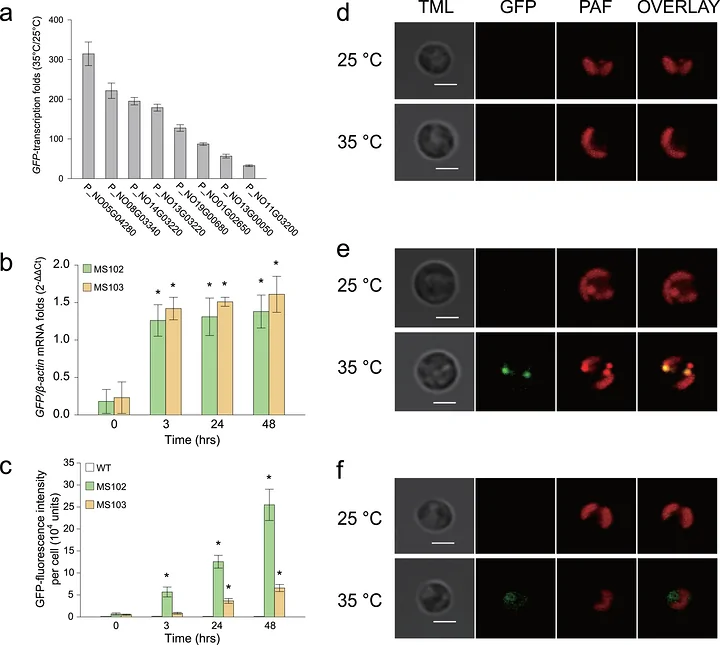
Identification of thermally inducible promoters from NoED and NoUK. (a) Fold changes of *gfp* transcripts driven by eight TOP thermal‐inducible promoter candidates. (b) *gfp* transcript levels in transgenic *N. oceanica* strains MS102 (harboring P*_NoED_*‐*gfp*) and MS103 (harboring P*_NoUK_*‐*gfp*) at 0, 3, 24, and 48 h under 35°C. (c) Quantification of GFP fluorescence (mean fluorescence intensity per cell) in WT, MS102, and MS103 strains at the indicated time points under 35°C. Data represent mean ± SD (*n* = 3). *p* ≤ 0.005 vs. WT. (d–f) Confocal images of WT (d), MS102 (e), and MS103 (f) after 48 h at 25°C or 35°C. These strains were also used for fluorescence quantification in panel (c). Green, GFP fluorescence; red, chlorophyll autofluorescence. TML, transmitted light; PAF, plastid autofluorescence. Scale bar, 2 µm.

To assess the regulatory performance of the identified promoters, we cloned each upstream of an enhanced GFP (eGFP) reporter and transformed *N. oceanica* via electroporation (Figure S3a). Transformants were grown under 25°C and shifted to 35°C for promoter induction. Eight promoters displayed strong and highly specific induction upon temperature elevation, with up‐regulation ranging from 30‐fold to more than 300‐fold (Figure 2a).

To quantify dynamic responsiveness, we monitored eGFP accumulation following variable heat‐pulse durations. All high‐performing promoters showed rapid induction kinetics, reaching half‐maximal activity within 2–4 h at 35°C, while reverting toward baseline levels within 24 h after temperature restoration. This reversibility highlights the suitability of these promoters for building a robust thermal “plug‐in” expression module that can be externally actuated and subsequently reset. Specifically, promoters *P_NO05G04280* and *P_NO08G03340* exhibited the highest induction strength, with minimal basal leakage at 25°C. Following heat stimulus, the transcriptional levels of *gfp* of the transformants MS102 (harboring *P_NO05G04280*, designated P*_NoED_*) and MS103 (harboring *P_NO08G03340*, designated P*_NoUK_*) exhibited a notable increase in expression within the first 3 h (6.1‐fold and 5.2‐fold, respectively) and remained relatively constant thereafter (Figure 2b).

Correspondingly, GFP fluorescence intensity increased by 36.5‐fold in MS102 and 11.7‐fold in MS103 maximally (Figure 2c). Fluorescence microscopy confirmed promoter‐driven expression at the single‐cell level (Figure 2d–f; WT, Figure 2d; MS102, Figure 2e; MS103, Figure 2f). Collectively, these results establish a set of native promoters with strong, specific, and tunable thermal inducibility that can be used as building blocks for a heat‐responsive expression system.

### 2.3 Construction of a Thermal Inducible “PLUG‐IN” Gene Module

**FIGURE 3:**
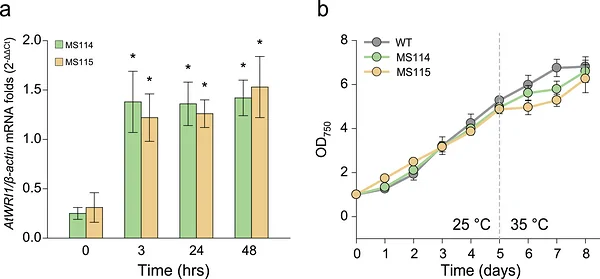
Heat‐induced expression of *AtWRI1* driven by P*_NoED_*‐ and P*_NoUK_* in *N. oceanica*. (a) *AtWRI1* transcript levels in transgenic strains MS114 (P*_NoED_*‐*AtWRI1*) and MS115 (P*_NoUK_*‐*AtWRI1*) at 0, 3, 24, and 48 h under 35°C. (b) Growth kinetics of WT, MS114, and MS115 at 25°C or 35°C. Data represent mean ± SD (*n* = 3). ^*^ *p* ≤ 0.005 vs. WT.

Based on promoter performance, P*_NoED_* and P*_NoUK_* were selected to construct a two‐component thermal “PLUG‐IN” module consisting of (i) a strong thermal‐on promoter and (ii) a downstream functional gene module. Each promoter was integrated into a defined genomic safe‐harbor site to ensure consistent expression across independent transformants. Using an oleaginous transcription factor, AtWRINKLED1 (AtWRI1), we demonstrated that the PLUG‐IN module supports programmable expression levels by modulating temperature duration and amplitude (Figure 3a). A short 3‐h pulse at 35°C activated expression but maintained subtle growth burden (Figure 3b), while extended induction maintained continuous amplification (Figure 3a). Specifically, *AtWRI1* transcriptional levels showed a continuous increase, with 5.5‐fold and 3.9‐fold increases in the positive lines MS114 (P*_NoED_*) and MS115 (P*_NoUK_*) (Figure 3a). Importantly, long‐term cultivation experiments showed that the temperature‐responsive expression system imposes negligible metabolic burden, preserves growth rates, and retains inducibility over at least 10 generations (Figure 3b; approximately 20 h per generation for *N. oceanica*). These results demonstrate that the chassis is genetically stable and operationally robust.

### 2.4 Thermal Regulation of Bioproduction Pathways

**FIGURE 4:**
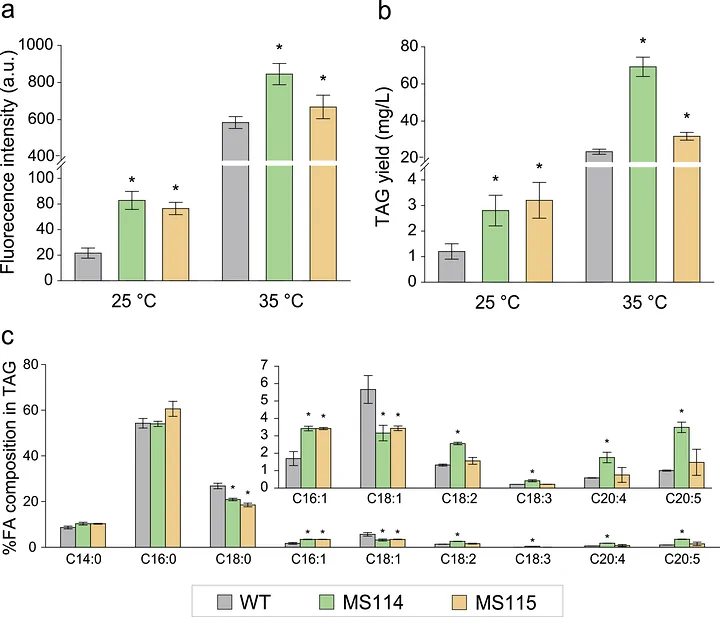
Heat‐induced oil accumulation mediated by P*_NoED_*‐ and P*_NoUK_* in *N. oceanica*. Nile Red fluorescence (a), TAG yield (b), and TAG‐associated fatty‐acid profiles (c) were quantified in WT, MS114, and MS115 cultured at 25°C or 35°C. Data represent mean ± SD (*n* = 3). ^*^ *p* ≤ 0.005 vs. WT.

To demonstrate practical utility, we applied the thermal PLUG‐IN chassis to assess the metabolic dynamics following the transfer from 25°C to 35°C. Cultures grown at 25°C displayed basal lipid levels, whereas a shift to 35°C triggered a marked increase in neutral lipid accumulation as measured by Nile Red staining and GC‐MS (Figure 4a,b). TAG content increased by 194% and 35% after 48 h induction. We also observed a degree of transcriptional leakage in the transformants under 25°C (Figure 4a,b). However, no significant loss of inducibility or increase in leakage and growth difference were observed after over 10+ generations, showing functional separation of growth and production phases.

Interestingly, the engineered strain MS114 (P*_NoED_*) exhibited significant changes in the composition of TAG‐associated PUFAs. Specifically, the content of key PUFAs increased by 2.5‐fold for C20:5, 2.1‐fold for C20:4, 26.4‐fold for C18:3, and 93% for C18:2 (Figure 4c), suggesting a reprogramming of lipid metabolism. These results validate the ability of the thermal chassis to control the timing and magnitude of bioproduction outputs.

### 2.5 Conditional Cell Wall Fragilization via *CesA1* Suppression

**FIGURE 5:**
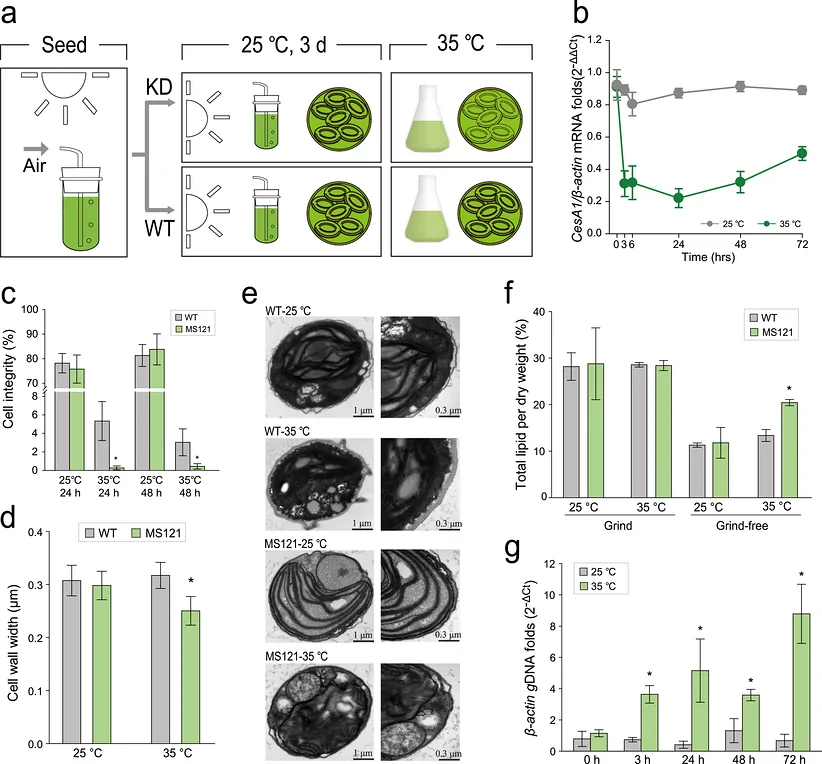
Thermal‐inducible cell‐wall weakening and permeability enhancement in *N. oceanica*. (a) Experimental design: the MS121 line was cultured at 25°C for 3 days and then shifted to 35°C for 24 or 48 h. WT served as the control. (b) *CesA1* transcript fold‐change between MS121 and WT at 0, 3, 6, 24, 48, 72 h under 25°C and 35°C. The value is calculated by 2^−[MS121(Ct(^ *^CesA1^* ^)‐Ct(^ *^β‐actin^* ^))/ WT(Ct(^ *^CesA1^* ^)‐Ct(^ *^β‐actin^* ^))]^. (c–e) Cell integrity (c), transmission electron microscopy images (d) and cell‐wall thickness (e) in WT and MS121 at 25°C or 35°C. Data represent mean ± SD (*n* = 3). ^*^ *p* ≤ 0.005 vs. WT. (f) *β‐actin* transcript fold‐change in MS121 and WT supernatant at 0, 3, 24, 48, 72 h under 25°C and 35°C. The value is calculated by 2^Ct(WT)‐Ct(MS121)^. Data represent mean ± SD (*n* = 3). ^*^ *p* ≤ 0.005 vs. 25°C. (g) Total lipid content in WT and MS121 at 25°C or 35°C. Data represent mean ± SD (*n* = 3). ^*^ *p* ≤ 0.005 vs. WT.

*N. oceanica* strains were engineered to express P*_NoED_*‐driven RNA interference (RNAi) cassette targeting cellulose synthase gene *CesA1*, which is essential for the biosynthesis of cellulose—the major component of the algal cell wall [27] (strain MS121; Figure S3c). Engineered *N. oceanica* strains in log‐phase were placed at 35°C for 24 or 48 h (Figure 5a). The transcription level of *CesA1* in MS121 at 35°C decreased by 94.6% on average compared to that at 25°C (Figure 5b). Under these conditions, promoter activity increased rapidly, with a detectable decline in cell viability within 24 h (Figure 5c**;** a 95% decline). Ultrastructural analysis of the cell wall revealed a 11.7% reduction in thickness for MS121 at 35°C, whereas the WT cell wall thickness remained unchanged (Figure 5d,e). No unintended induction occurred at 25°C controls, confirming that the potential basal leakage at the transcriptional level was negligible and the thermal‐activated module is competent in controlling the cell wall fragilization system (Figure 5d,e).

To further evaluate the nutrient extractability of the P*_NoED_*‐*CesA1* RNAi construct, both MS121 and WT strains were cultured to late‐log phase under two temperature conditions: 25°C and 35°C. MS121 released 1.7‐ to 11.9‐fold more DNA into the culture medium under 35°C than under 25°C (Figure 5f), suggesting uneven reduction in the thickness of the cell wall and a degree of cell lysis of MS121. The successful validation of the conditional cell‐wall‐fragilization module has inspired us to explore the potential application of this module for lipid accessibility without the process of cell grinding. Lipid content was assessed with and without cell grinding (see Methods). Under 35°C, the lipid yield from MS121 was significantly higher, increasing by 53% compared to the WT, even without cell grinding (Figure 5g). This suggests enhanced extractability of intracellular compounds in MS121, likely due to weakening of the cell wall. In contrast, when cells were ground prior to lipid extraction, no significant difference in lipid yield was observed between MS121 and WT under 35°C. Lipid yields remained comparable between MS121 and WT under normal conditions at 25°C, regardless of whether cell grinding was performed (Figure 5g).

These findings confirm the efficacy of the P*_NoED_*‐*CesA1* RNAi construct in inducing thermally controlled cell wall fragilization in *N. oceanica*. This inducible system allows engineered cells to maintain normal growth under standard conditions while enabling controlled fragilization and leakage of cellular components under heat stress, providing a valuable platform for applications in biotechnology.

### 2.6 The PLUG‐IN Chassis is Activatable by Mammalian Body Temperature

To test whether the thermal PLUG‐IN system could be activated by naturally occurring biological heat sources, we evaluated its responsiveness to mammalian body temperatures using small and large warm‐blooded animals. Because vertebrate body temperatures (mice, 36°C–37 °C; rabbits, 37°C–39°C) closely match the induction threshold of the promoters identified above, these systems provide an ideal test bed for real‐world thermal actuation.

**FIGURE 6:**
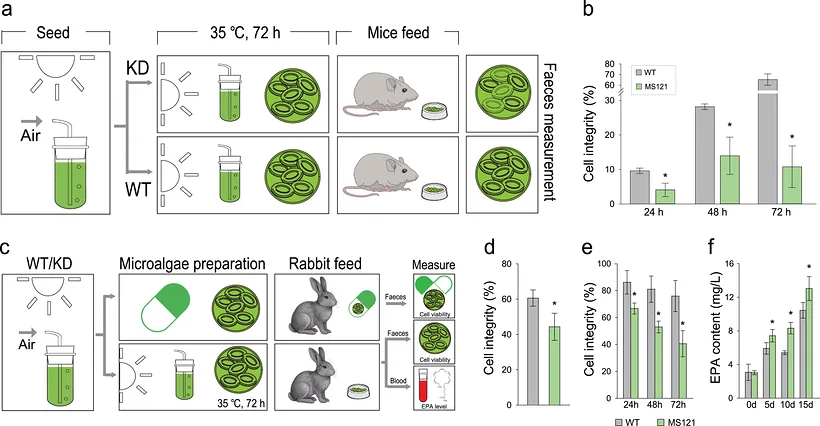
Thermal‐induced cell‐wall fragilization enhances mammalian bioaccessibility of *N. oceanica*. (a) Experimental design: MS121 (KD) was cultured at 25°C for 3 days and then administered daily to mice with equivalent algal cells (2 × 10^9^); WT served as the control. (b) Cell integrity of WT and MS121 recovered from mouse faeces. (c) Experimental design: KD was cultured at 25°C for 3 days and then administered daily to rabbits; WT served as the control. (d) Cell integrity of WT and MS121 recovered from capsules in rabbit faeces. (e) Cell integrity of WT and MS121 recovered from rabbit faeces. (f) EPA levels in the blood of rabbits fed with WT or MS121 with equivalent algal cells (2 × 10^9^). Data represent mean ± SD (*n* = 3). ^*^ *p* ≤ 0.005 vs. WT.

To test this hypothesis, both MS121 and WT with equivalent cell numbers were fed to mice for three consecutive days (Figure 6a). Fecal samples were collected daily, resuspended, and analyzed for cell viability microscopically. The results demonstrated a pronounced decline in MS121 cell viability relative to WT. Specifically, MS121 exhibited 58%, 50%, and 84% reductions in viability after 24, 48, and 72 h, respectively (Figure 6b). These results reveal that heat‐inducible cell wall weakening in MS121 substantially enhances its susceptibility to mice digestion, thereby potentially improving nutrient release and laying the foundation for its application as a more bioavailable algal feed and functional food source.

To evaluate performance under higher physiological temperatures, we conducted parallel experiments in rabbits. To avoid potential confusion caused by bacteria or other particles in the feces, capsule feeding and blood analysis were conducted (Figure 6c). The capsules were excreted by rabbits after approximately 12 h, and the cell viability of MS121 decreased by 30% compared with the WT (Figure 6d). In parallel, without a capsule, cell viability of MS121 decreased by 23%, 36%, and 48% relative to the WT, after 24, 48, and 72 h, respectively (Figure 6e).

Since EPA is a hallmark PUFA in *N. oceanica*, we measured its level in rabbit blood as an indicator of MS121/WT nutrient absorption. The EPA content in the blood of rabbits fed with MS121 was 20%, 40%, and 44% higher than that of rabbits fed with WT, after 5, 10, and 15 days of feeding, respectively (Figure 6f). Therefore, the thermal PLUG‐IN chassis functions as a biologically compatible, externally triggerable expression system that can be activated reliably by mammalian physiological heat. This expands its potential applications to include in vivo biosensing, responsive live biomaterials, and thermally actuated symbiotic biotechnologies.

### 2.7 Environmental Robustness of the PLUG‐IN Chassis

To verify that the engineered heat‐inducible system remains robust under practical cultivation scenarios, we monitored the growth kinetics and physiological vigor of MS121 vs. WT over an 18‐day period. By implementing a daily 1‐h heat pulse (35°C) to mimic outdoor temperature shifts, we found that MS121 not only avoided the growth inhibition typically associated with engineered strains but actually outperformed the WT. Specifically, MS121 showed a 7.2% increase in OD_750_ and a 32.5% increase in cell density (Figure S4a,b), while maintaining physiological parameters (*F*v/*F*m and cell size) comparable to the WT (Figure S4c,d). This superior performance, potentially driven by accelerated cell division via modified cell wall integrity, highlights MS121 as a promising candidate for industrial‐scale applications.

## 3 Discussion

The primary innovation of this study is the development of a versatile, thermally‐inducible “PLUG‐IN” chassis in the promising synthetic biology chassis *N. oceanica*, which we validated with two distinct biotechnological applications: high‐value lipid production and enhanced mammalian digestibility. This platform is built on the novel, strong promoter P*_NoED_* and provides a powerful tool for uncoupling robust biomass cultivation from the expression of target payloads. We demonstrated the chassis's utility as a cell factory by “plugging in” the master regulator *AtWRI1*. We further demonstrated the platform's utility by “plugging in” a *CesA1‐RNAi* cassette. While Matsui [27] previously reported a constitutive *CesA* knockout, that approach suffers from a critical growth‐lysis trade‐off, rendering the strain fragile during cultivation. Our inducible “PLUG‐IN” system is fundamentally superior as it solves this trade‐off. The MS121 chassis grows robustly like wild‐type but can be triggered to fragilize its cell wall synthesis on command.

This “PLUG‐IN” chassis is not limited to lipids or digestibility. Its nature as a general‐purpose, controllable photosynthetic platform makes it ideal for producing a wide array of other high‐value compounds, such as pharmaceuticals, nutraceuticals, or complex proteins, where inducible expression is paramount. Although direct transcriptional activation in the gastrointestinal tract remains to be examined, the thermal‐inducible feature, operating at a physiologically relevant 35°C, makes the chassis uniquely suited for applications in functional foods and animal feed, as demonstrated by our in vivo mammalian experiments where the *CesA1* plug‐in led to 44% higher absorption of the chassis's native high‐value product (EPA). A combination of the conditional‐cell‐wall‐fragilization “PLUG‐IN” chassis with other functional modules, such as pharmaceutics procudtion, would further increase the value of this photosynthetic cell factory.

The advantage of our system lies in temporal decoupling of growth and production, rather than absolute repression. We acknowledge that the engineered strains exhibited measurable basal expression of target genes even at the permissive temperature (25°C), as reflected in the elevated TAG levels in the “off” state. However, no significant loss of inducibility or increase in leakage and no growth difference were observed after over 10+ generations. Moreover, no unintended induction of cell wall fragilization occurred at 25°C controls, confirming that the potential basal leakage at the transcriptional level might be negligible. While such promoter leakage is a common feature of many inducible systems, its implications for industrial application must be carefully considered. In a production setting, even low‐level constitutive expression could impose a metabolic burden during the growth phase or lead to the emergence of “escapee” mutants that outcompete the engineered population over prolonged cultivation. To mitigate this, future iterations of the PLUG‐IN chassis could incorporate genetic inverters, dual‐control circuits, or promoter engineering to further reduce basal activity. Nevertheless, the present system already achieves a clear functional separation between growth and production phases, allowing biomass accumulation without severe fitness costs—a key advantage over strong constitutive expression systems.

The use of 35°C as an induction threshold, while effective for mammalian‐thermal triggering, raises practical questions regarding environmental robustness in outdoor cultivation systems. In many tropical or temperate regions, open pond temperatures can naturally exceed 35°C during summer months, potentially leading to unintended induction. To ensure reliable control in scalable bioprocesses, we propose several strategies: (i) employing shaded or actively cooled cultivation systems to maintain sub‐induction temperatures during biomass accumulation; (ii) utilizing enclosed photobioreactors for precise thermal management; or (iii) developing engineered strains with higher thermal activation thresholds through promoter evolution or orthogonal regulatory circuits. Such adaptations would allow the PLUG‐IN system to be deployed in a variety of climatic conditions without compromising control over production timing. From a bioprocess perspective, the ability to induce cell wall weakening on‐demand could significantly reduce downstream energy inputs for cell disruption, thereby improving the overall energy balance and economic feasibility of microalgal biorefineries. Future work will focus on integrating the PLUG‐IN chassis with existing cultivation infrastructure and evaluating its performance under real‐world seasonal temperature variations.

## 4 Materials and Methods

*N. oceanica* IMET1 was cultivated in modified f/2 medium under continuous light (50 ± 5 µmol photons m^−^^2^ s^−^^1^) at 25°C, with HS induced by transferring cultures to 35°C. Outdoor simulation was conducted under 25°C with incubation under 35°C for 1 h at a fixed time point each day. Transcriptomic profiling was performed at 0, 3, 24, and 48 h, and differentially expressed genes were identified based on ≥2‐fold change with false discovery rate–corrected *p* < 0.05. Candidate promoter sequences (1 kb upstream of start codons) were cloned and fused to *GFP* or *AtWRI1* in expression vectors, and transformed into *N. oceanica* via high‐voltage electroporation. Transgenic lines were confirmed by PCR and Sanger sequencing, with expression levels quantified by qRT‐PCR, fluorescence microscopy, and lipid analysis via TLC and GC‐MS. For *CesA1* knockdown, an RNAi cassette driven by the thermal‐inducible promoter P*_NoED_* was constructed, and cell wall morphology was examined by transmission electron microscopy. Levels of *CesA1* and *β‐actin* were quantified by qRT‐PCR. Primers are provided in Table S1. Lipid yields were measured with or without cell disruption. Digestibility was tested by feeding engineered strains (MS121) or wild‐type to C57BL/6 mice and Japanese White rabbits, followed by fecal cell viability assays and EPA quantification in rabbit blood. Statistical analyses were performed in triplicate using one‐way ANOVA. Detailed protocols, sequences, and transcriptomic data are provided in Supporting Information Appendix.

## Author Contributions

Q.G., Y.X. and Y.L. designed research; Y.X. and S.W. constructed the plasmids; Z.L. and Q.W. performed the animal and microscopic experiments; Q.G., Y.X., Y.C., T.X., X.C., X.H. and C.L. generated and screened transgenic lines of *N. oceanica*; M.D., C.M. and Y.C. conducted the GC‐MS assay; Y.X. performed phylogenetic analysis; Q.G., Y.X. and Y.L. analyzed data and wrote the paper.

## Funding

This work was financially supported in part by grants from the National Key R&D Program of China (2021YFA0909600), the National Natural Science Foundation of China (32560020 and 32370380), the Key R&D Program of Hainan Province (ZDYF2024XDNY244), the Foreign Expert Foundation of Hainan Province (G20230607016E), and the Hainan Tropical Ocean University Joint Open Project for Aquatic South Breeding (2023SCNFKF04).

## Conflicts of Interest

The authors declare no conflicts of interest.

## Supporting information




**Supporting File 1**: advs76102‐sup‐0001‐SuppMat.docx.

**Supporting File 2**: advs76102‐sup‐0002‐figures and tables.pdf.

## Data Availability

The data that support the findings of this study are available on request from the corresponding author. The data are not publicly available due to privacy or ethical restrictions.
