# Supplementary material for: A Modular PLUG‐IN Photosynthetic Chassis With Tunable Thermal Control for Mammalian Systems
Source: Adv Sci (Weinh). 2026 Jun 15;13(50):e76102. doi: 10.1002/advs.76102 (PMC13336961; doi:10.1002/advs.76102)
Supplement: Supplementary file 1 [file ADVS-13-e76102-s001.pdf]

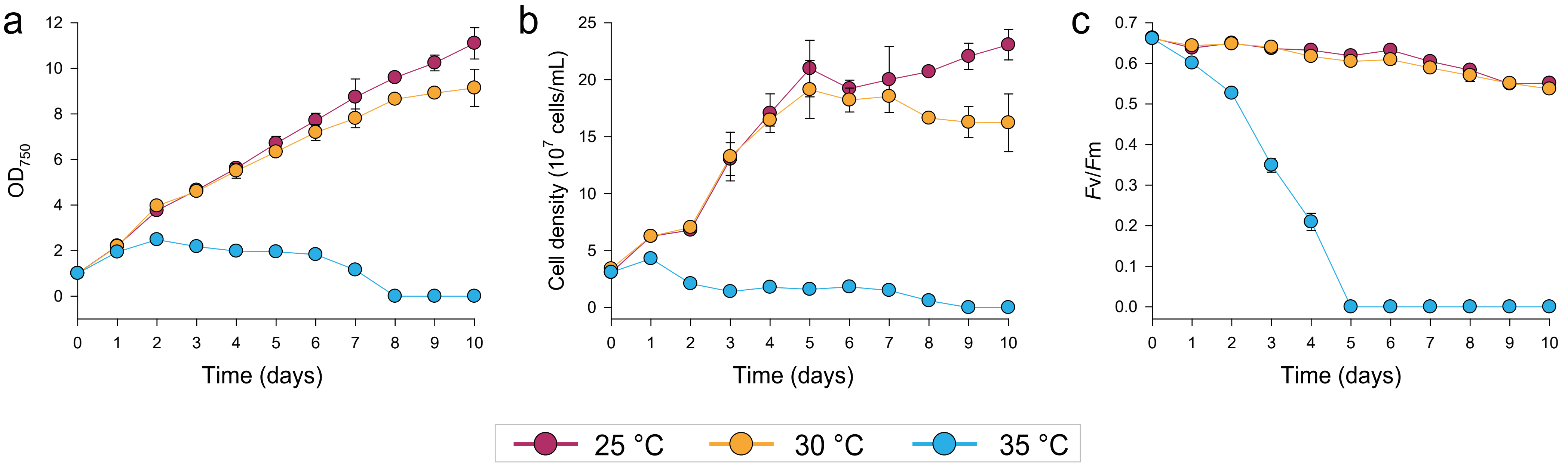

Fig. S1 Growth kinetics of *N. oceanica* under 25 °C, 30 °C and 35 °C. OD_{750}(**a**), cell density (**b**) and F_v/F_m (**c**) were measured over 10 days.

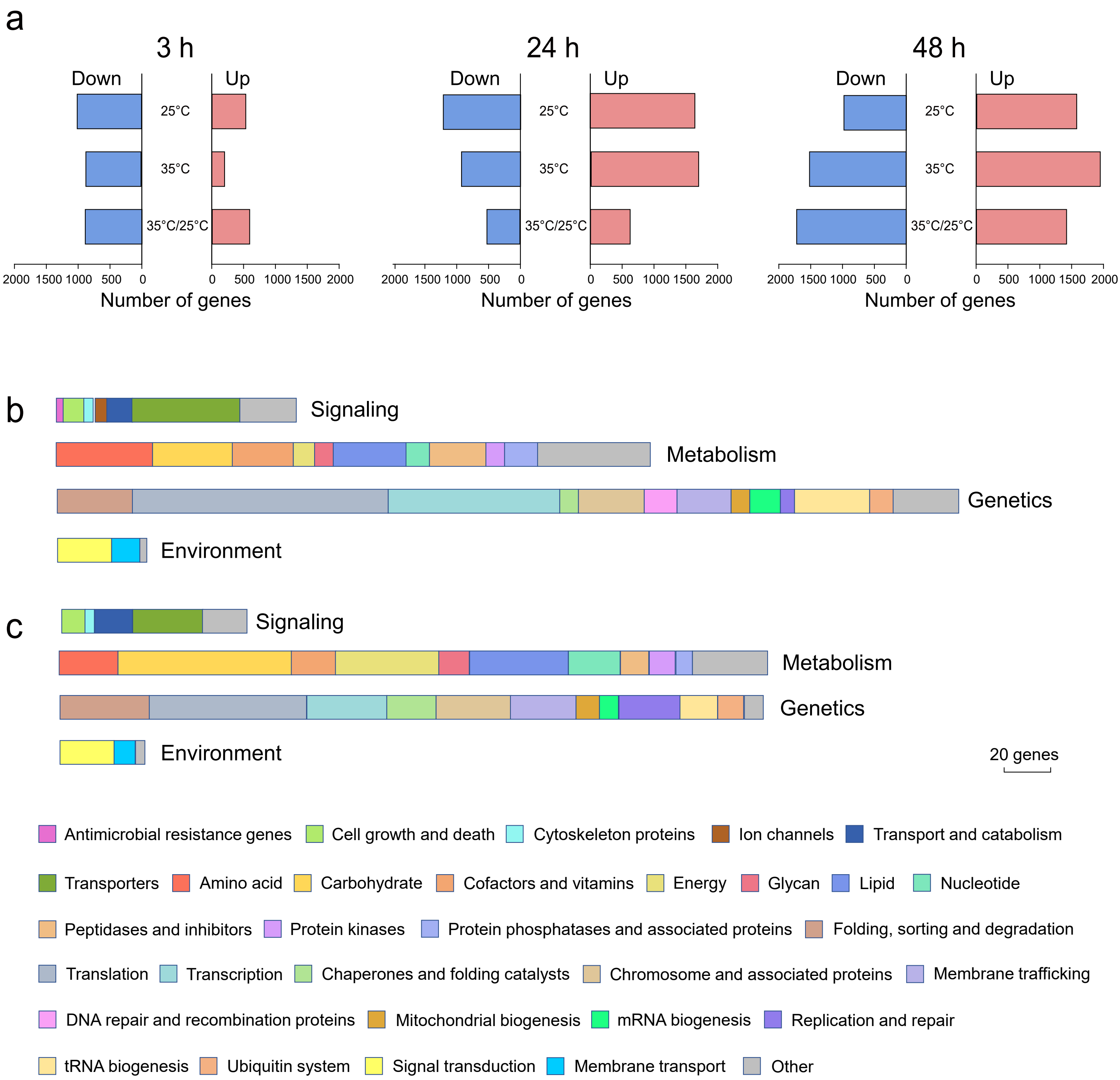

Fig. S2 Transcriptomic comparison between 25 °C and 35 °C in *N. oceanica*. The transcriptomics were measured and compared at 48 h under 25 °C and 35 °C. (**a**) Number of up- or down-regulated genes at each of the time points under 25 °C (left; relative to 0 h), 35 °C (middle; relative to 0 h) and 35 °C/25 °C (right). (**b-c**) Distribution of genes in manual functional categories within each cluster was caculated to elucidate up-regulating (**b**) and down-regulating (**c**) genes. Bar length indicates gene munbers among various functional categories.

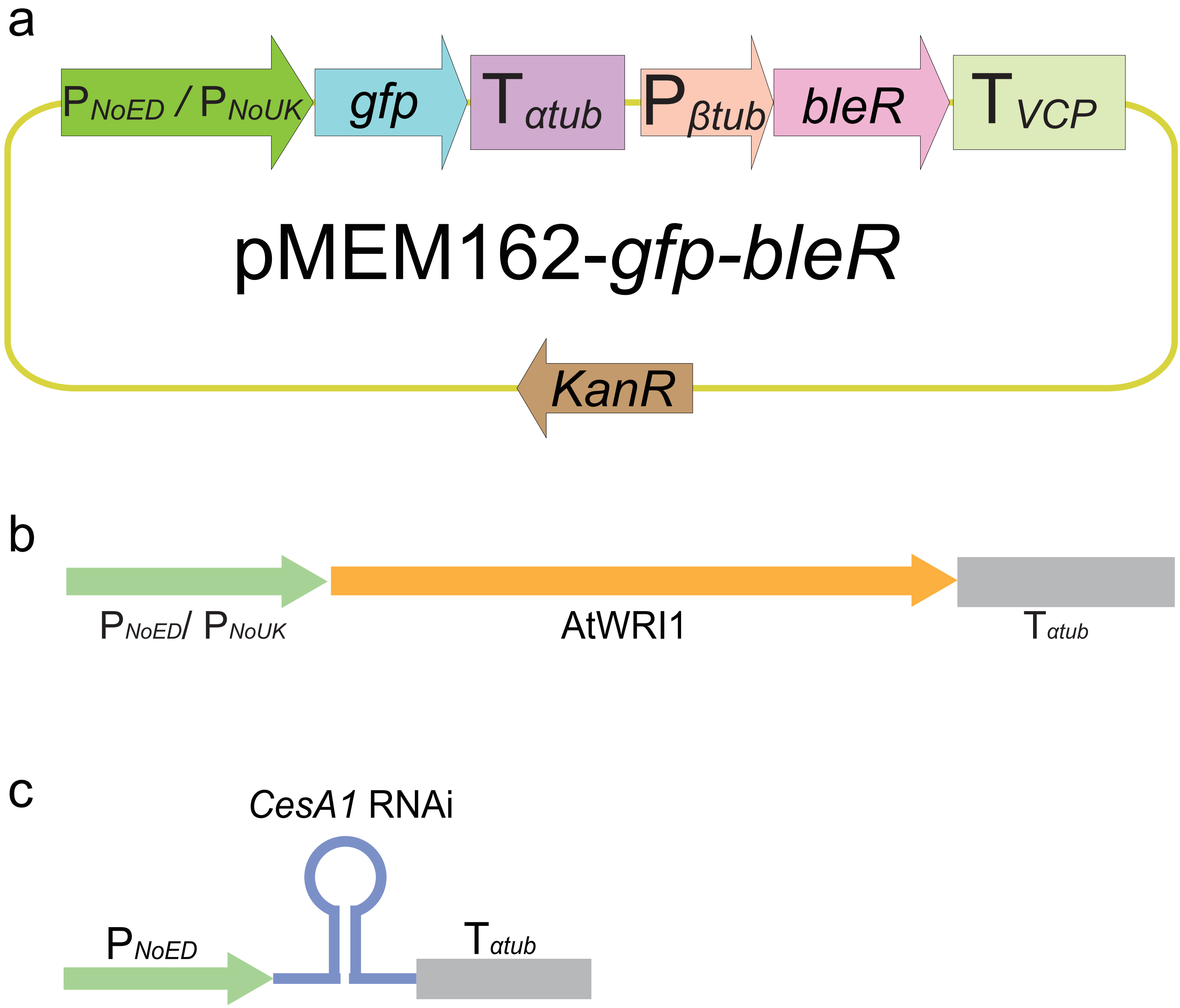

Fig. S3 Map of vectors for (**a**) expression of green fluorescent protein (GFP) by *NoED* promoter (P_{NoED}) or *NoUK* promoter (P_{NoUK}), (**b**) expression of *Arabidopsis thaliana* WRINKLED1 (AtWRI1) by P_{NoED} or P_{NoUK}, and (**c**) RNAi knockdown of cellulose synthase genes *CesA1* expression by P_{NoED}. $P_{\beta tub}$, promoter of *β-tublin*; *bleR,* zeocin resistance gene; *KanR*, kanamycin resistance gene; $T_{\alpha tub}$, terminator of α-tublin; T_{vcp}, terminator of violaxanthin/chlorophyll a binding protein (VCP).

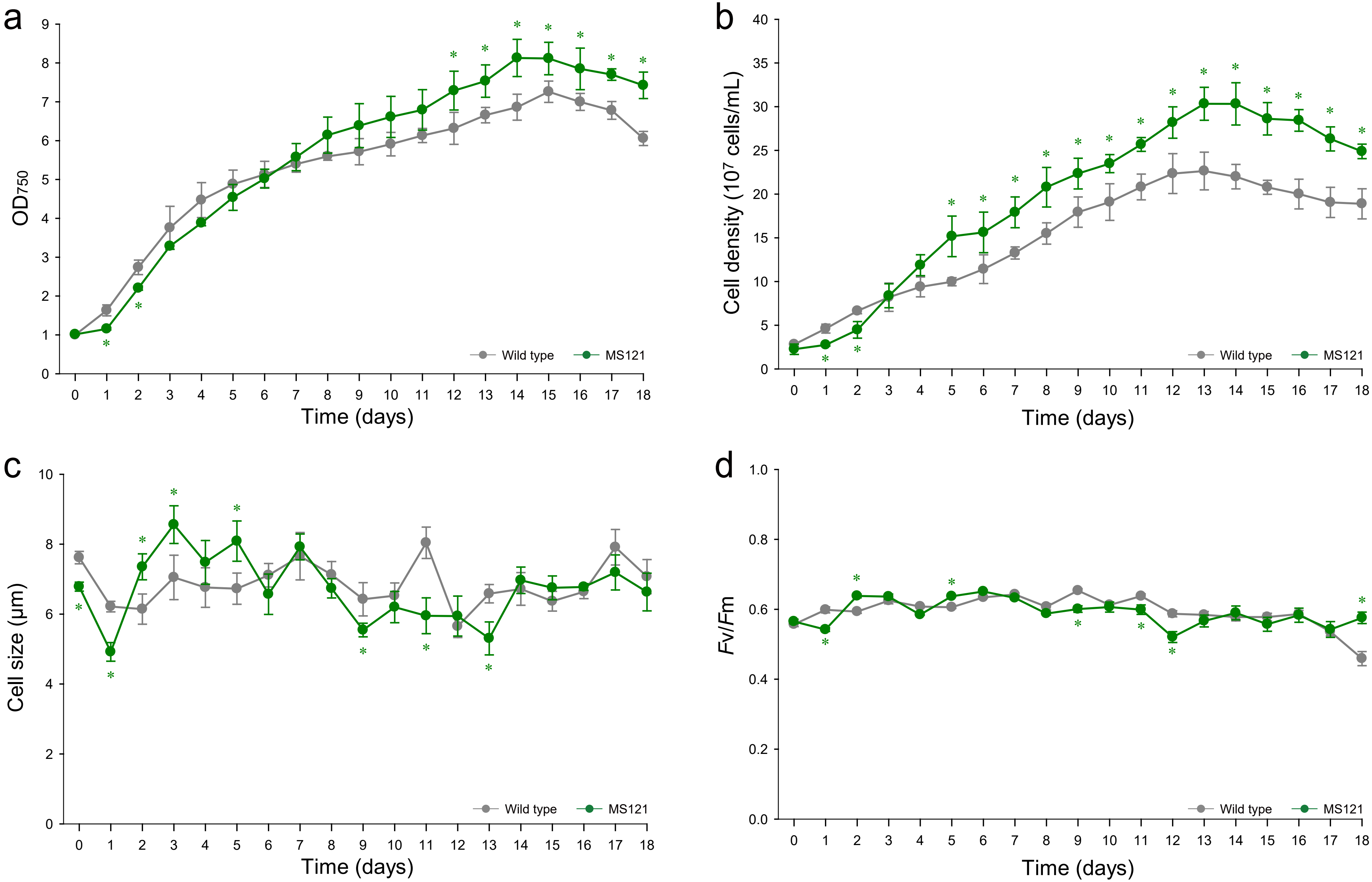

Fig. S4 Comparison of simulative outdoor kinetics between MS121 and *N. oceanica* wild type. To investigate whether the MS121 strain exhibits maladaptation under outdoor conditions, we conducted a simulated outdoor experiment. After synchronizing the OD_{750} of MS121 and wild type (WT) to 1.0, the cultures were maintained at 25 °C for 18 days. At a fixed time point each day, both MS121 and WT were incubated at 35 °C for 1 hour. (a-d) OD_{750} (a), cell density (b) , cell size (c) and *F*v/*F*m (d) in WT and MS121. Data represent mean ± SD (n = 5). *$p \leq 0.005$ versus WT.

Table S1. Primers used in this study.

	Forward primer (5' to 3')	Reverse primer (5' to 3')
**Construction of *GFP* plasmids to identify thermal-inducible promoters**		
P_{NoED}	CAGTGGTGGTGGTGGAAGCTTTAATGCACTAGTTTTCGGATGT	CTCGCCCTTGGCCATGGTACCCGCCGGCAGCCGAGTCGGTG
P_{NoUK}	CAGTGGTGGTGGTGGAAGCTTCTCGAAATAACACGGGCTAGA	CTCGCCCTTGGCCATGGTACCCCTATTGCCTCTAGCCAGTG
**Construction of *AtWRI1* plasmids to validate thermal-inducible promoters**		
P_{NoED}	ACTCGGCTGCCGGCGGGTACCATGAAGAAGAGATTGACTACTTCT	TCCCTCCTCCTGGGATCCTTAAACAAGATAATTACAAGA
P_{NoUK}	GCTAGAGGCAATAGGGGTACCATGAAGAAGAGATTGACTACTTCT	TCCCTCCTCCTGGGATCCTTAAACAAGATAATTACAAGA
**Construction of *CesA1* RNAi plasmid**		
CesA1-long	CGCGGCGGGCGCCCCCGCGGGA	CATCGTCTCGGCAATAATAAAG
CesA1-short	CGCGGCGGGCGCCCCCGCGGGA	ACAGCGAGACCTCGTCCGCCACCG
**Transformant validation**		
pMEM188	AATATACGTGGTAATGAGGAGAG	CTGCTTCATGTGGTCGGGGTAGC
pMEM190	CTGCTTTATTGGCCCACTC	CTGCTTCATGTGGTCGGGGTAGC
pMEM195	AATATACGTGGTAATGAGGAGAG	CAAGTTCCTTAGTATAAG
pMEM196	CTGCTTTATTGGCCCACTC	CAAGTTCCTTAGTATAAG
pMEM197	GTGCTACGTCTGTCGACAGG	GACAGGTGAGGACTTGAAGG
**RT-qPCR**		
GFP	CCGGCGAGGGTGAGGGTGACGC	GGTGTCGCCCTCGAACTTGACC
AtWRI1	CTGAAGCTCCTAGACCTAAGAGAGC	GCAGCAAGATCATAAGTATGAG
CesA1	GCCCATCGCCTCCTCAGTCGC	CGTGGATTTTCTGCTTGAGGGTGG
β-actin	GACGGCACCAAGGTCAAAAT	ACGACGTGGAAGAGGAGGAA
