# Supplementary material for: A Modular PLUG‐IN Photosynthetic Chassis With Tunable Thermal Control for Mammalian Systems
Source: Adv Sci (Weinh). 2026 Jun 15;13(50):e76102. doi: 10.1002/advs.76102 (PMC13336961; doi:10.1002/advs.76102)
Supplement: Supplementary file 2 [file ADVS-13-e76102-s002.docx]

**Strains and culture conditions**

*Escherichia coli* strain transetta (DE3) was grown in Luria–Bertani (LB) medium at 37 °C with shaking at 200 rpm. *N. oceanica* IMET1 was cultivated in modified f/2 liquid medium containing 35 g/L sea salt, 1000 mg/L NaNO_3_, 66.6 mg/L NaH_2_PO_4_·H_2_O, 3.65 mg/L FeCl_3_·6H_2_O, 4.37 mg/L Na_2_EDTA·2H_2_O, 0.0196 mg/L CuSO_4_·5H_2_O, 0.0126 mg/L Na_2_MoO_4_·2H_2_O, 0.044 mg/L ZnSO_4_·7H_2_O, 0.0109 mg/L CoCl_2_·6H_2_O, 0.036 mg/L MnCl_2_·4H_2_O, 5 µg/L VB_12_, 5 µg/L biotin and 0.1 mg/L thiamine HCl (1-3). Cells were cultivated in liquid cultures under continuous light (approximately 50 ± 5 µmol photons m^-2^ s^-1^) at 25 °C. For thermal induction, the *N. oceanica* cells were harvested by centrifugation (3500 × *g* for 5 min) and then resuspended in the 35 ℃ f/2 medium. After switching to the designated culture condition, cell samples were taken at 3 h, 24 h and 48 h from each column for transcriptomic profiling. Cell growth was determined based on both cell density and optical density (OD_750_).

To characterize the transgenic lines, mid-logarithmic phase algal cells (OD_750_ of 2.6) were collected for validating successful transformants via PCR amplification and Sanger sequencing. Positive lines were then cultured for further measurements. For phenotyping, the transgenic lines (plus the WT control) were grown to OD_750_ of 4.5±0.5 for five days, and then the TAG content and TAG-associated FA profiles were tracked at 48 h under either 25 ℃ or 35 ℃.

To evaluate the practical applicability of MS121 in real-world production, MS121 and *N. oceanica* WT were cultured separately for 18 days at 25 °C under 50 ± 5 µmol photons m⁻² s⁻¹. At a fixed time each day, the cultures were transferred to 35 °C for 1 h. Growth performance, including OD_750_, cell density and cell size, as well as photosynthetic efficiency (*F*v/*F*m), was monitored daily.

**Identification of key genes that respond to heat stress and their promoters**

To identify the heat-responsive genes in *N. oceanica* IMET1, gene expression over 0 h, 3 h, 24 h and 48 h was considered different if the FPKM value showed at least two-fold change while the false discovery rate (FDR)-corrected *p*-value was <0.05 (2). The promoter sequence was defined as the upstream 1 kb sequence relative to the translation start site of the gene as previous description.

**Cloning and phylogenetic analysis of genes**

*N. oceanica* genomic DNA was used as PCR templates. PCR products were then sequenced to obtain the full-length *NoED* protein-coding sequences. In addition, the extracted genomic DNA was used as template to amplify the genomic *NoED* that include both introns and exons. Then the gene structure (i.e., exons, introns, and untranslated regions) was verified by alignment and comparison between the protein sequence and the genome sequence.

The encoded protein sequence of *NoED* were aligned with MUSCLE version 3.8.31 (4) and adjusted manually using BioEdit version 7.0.5.3 (5) before phylogenetic analysis. The optimal substitution model of amino acid substitution was selected using ModelGenerator version 0.84 (6). The curated alignment was then used to construct a phylogenetic tree via the neighbor-joining (NJ) method in MEGA4.1 (7), with the tree tested by bootstrapping with 1000 replicates.

**Vector construction and *N. oceanica* transformation**

To validate the thermal-induced promoters in *N. oceanica*, a green fluorescent protein (GFP) reporter system was designed and constructed. Fourteen assumed thermal-induced promoters was one-by-one inserted into a backbone vector of pXJ53 (containing a core cassette of *gfp*-*α-tubulin* terminator-*β-tubulin* promoter-*ble*-*iolaxanthin/chlorophyll a binding protein* (*VCP*) terminator) to produce pMEM181-pMEM194, including pMEM188 (harboring P*_NoED_*) and pMEM190 (harboring P*_NoUK_*) (**Figure S3A**). To construct the vectors for *AtWRI1* expression in *N. oceanica* IMET1, *AtWRI1* cDNA were amplified and subcloned into pMEM188 and pMEM190, respectively, forming pMEM195 (harboring P*_NoED_*) and pMEM196 (harboring P*_NoUK_*), respectively (**Figure S3B**). To construct vector for cellulose synthase (CesA1) RNAi knockdown, a 200 bp small fragment (corresponding to the *CesA1* nucleotide sequence 540-739 bp) and a 400 bp long fragment (corresponding to *CesA1* gene sequence 340-739 bp) were amplified from the *N. oceanica* IMET1 cDNA, respectively. The fragments were digested with *Eco*RI and *Xba*I and joined with *Xba*I sites. The joint fragments with the inverted sequences were ligated to the *Eco*RI site of the linearized pXJ480 vector to create pXJ480-*CesA1* plasmid, respectively. P*_NoED_* was digested with *Sac*I and *Nco*I and ligated in the pXJ480-*CesA1* plasmid replacing the*β-tublin* promoter to form pMEM197 (**Figure** **S3C**).

Nuclear transformation of *N. oceanica* was performed for linearized vectors using the high-voltage (11,000 V/cm) electroporation method (8). Positive lines were identified by PCR and Sanger sequencing at first, then the transcription level of *gfp, AtWRI1* or *CesA1* were tested by qRT-PCR in isolated lines at 0, 3, 6, 24, 48, and 72 h under 35 ℃. *β-actin*, which is considered as identical transcription, was used as a control. In addition, to determine whether cellular contents leaked more readily from MS121 than from *N. oceanica* WT at 35 °C, 1.5 mL of culture supernatant was collected from MS121 and WT cultures after 0, 3, 24, 48, and 72 h of incubation at 35 °C. Genomic DNA (gDNA) was extracted from each supernatant sample, dissolved in 20 μL ddH_2_O, and subjected to qRT-PCR analysis of *β-actin* (**Table S1**). The relative gDNA leakage level of MS121 versus WT was then calculated and compared using the formula as 2^-[MS121(Ct(CesA1)-Ct(β-actin))/ WT(Ct(CesA1)-Ct(β-actin))]^.

**Fluorescence and Electron Microscopy**

Characterization of WT, MS102 and MS103 was carried out with a laser-scanning confocal microscope, FluoView FV1000 (Olympus, Japan). Chlorophyll autofluorescence was excited at 559 nm and detected at a bandwidth of 650-750 nm. Fluorescence of GFP was excited at 488 nm and detected at a bandwidth of 500-525 nm. GFP intensity was then quantified by Olympus Flowview Ver 4.0b. For each of the lines, GFP intensity of ten cells was quantified and the average intensity per cell was used for comparison across lines. The vital rates were measured by LUNA (Logos Biosystems, Korea).

Cells were prepared for electron microscopy as previously described. Briefly, cells were fixed in a 1:1 mixture of 2% OsO_4_ and 3% ferrocyanide (w/v), dehydrated in a graded ethanol series, and immersed and polymerized in 100% resin at 70℃. Polymerized sections were stained with 3% (w/v) uranyl acetate and Reynold’s lead citrate (9), and then examined and photographed under a JEM-1010 transmission electron microscope operated at 80 kV (JEOL, Tokyo, Japan). Images were recorded on Kodak EM film 4489 (Eastman Kodak Co., New York, NY, USA) and scanned to tagged image file (TIF) format using an Epson Perfection V700 photo scanner (Epson Korea Co., Ltd., Seoul, Korea). Cell wall thickness was analyzed using ImageJ2 (https://imagej.net/ImageJ); cell walls from six individual cells were measured for each sample.

**Lipid isolation and quantification via TLC and GC-MS**

Before extraction, all dried samples are grinded via 180 ms, 4.5 m/s vibration. Then the mixture is pooled into chloroform:methanol (2:1 [v/v]) for 2 h shaking under 100 rpm, 30 ℃. As for *CesA1* RNAi samples, the grinding step was canceled. Total lipids were extracted using chloroform:methanol (2:1 [v/v]) with 100 mM internal control of tri13:0 TAG and separated on a silica TLC plate using a mixture of solvents consisting of petroleum ether, ethyl ether and acetic acid (70:30:1, by volume). To quantify the amount of TAG accumulated in *N. oceanica* WT and transgenic lines, TAG bands were scraped from the TLC plate. Fatty acid methyl esters (FAMEs) were prepared by acid-catalyzed transmethylation of the TAG bands and then analyzed by GC-MS (10). Mixed analytical standards of FAMEs and pentadecane were used as external and internal standard, respectively. The amounts of TAGs and the profiles of TAG-associated FA were calculated based on the results derived from GC-MS. The chemicals used as standards were purchased from Sigma, USA.

**Animal**

C57BL/6 mice and Japanese White rabbits (SPF grade) were obtained from Haikou Yushi Biology Technological Co. Ltd. Animal were kept under standard conditions of 12 h night–day cycle, 23 ± 2-3°C and controlled humidity. After 1 week of acclimatisation, mice were fed with WT or MS121 for three days, with daily measurements of faeces for the cell viability. To be specific, the feeding algal solution was prepared with a cell density of 2.00 × 10⁹/mL. Fecal samples were selected to be as green and fresh as possible. Each 0.3 g of fecal sample was treated with 1 mL of PBS buffer solution, thoroughly mixed, and subsequently diluted 10 times for further analysis. Cell integrity was measured by LUNA (Logos Biosystems, Korea). Blood EPA level was measure according to below lipid isolation and quantification. All animal procedures were performed in accordance with the guidelines of China. Approval for this animal study was granted by Hainan University Application for Animal Welfare and Ethical Committee (application No. *HNUAUCC-2025-00002*).

**Statistical analysis**

All experiments were in triplicates, with results presented as mean ± standard deviation (SD). Statistical analysis was performed using Graphpad Prism 5 (GraphPad, USA). The *p*-values were calculated via one-way analysis of variance (ANOVA).

**Data Availability**

The sequences of NoED and NoUK are deposited in NanDeSyn under NO05G04280 and NO08G03340 respectively. The transcriptomic data are deposited in NanDeSyn (nandesyn.single-cell.cn). The coding sequences of AtWRI1 are deposited in GenBank under NP_001325502.1.

**Reference**

1.	J. Jia *et al.*, Molecular mechanisms for photosynthetic carbon partitioning into storage neutral lipids in *Nannochloropsis oceanica* under nitrogen-depletion conditions. *Algal Res* **7**, 66-77 (2015).

2.	J. Li *et al.*, Choreography of transcriptomes and lipidomes of Nannochloropsis reveals the mechanisms of oil synthesis in microalgae. *Plant Cell* **26**, 1645-1665 (2014).

3.	A. Moustafa *et al.*, Genomic footprints of a cryptic plastid endosymbiosis in diatoms. *Science* **324**, 1724-1726 (2009).

4.	R. C. Edgar, MUSCLE: a multiple sequence alignment method with reduced time and space complexity. *BMC Bioinformatics* **5**, 113 (2004).

5.	T. Hall, BioEdit: A user-friendly biological sequence alignment program for Windows 95/98/NT. *Nucleic Acids Symp Ser* **41**, 95-98 (1999).

6.	T. M. Keane, C. J. Creevey, M. M. Pentony, T. J. Naughton, J. O. McLnerney, Assessment of methods for amino acid matrix selection and their use on empirical data shows that ad hoc assumptions for choice of matrix are not justified. *BMC Evol Biol* **6**, 29 (2006).

7.	K. Tamura, J. Dudley, M. Nei, S. Kumar, MEGA4: Molecular Evolutionary Genetics Analysis (MEGA) software version 4.0. *Mol Biol Evol* **24**, 1596-1599 (2007).

8.	Q. Wang *et al.*, Genome editing of model oleaginous microalgae *Nannochloropsis* spp. by CRISPR/Cas9. *Plant J* **88**, 1071-1081 (2016).

9.	E. S. Reynolds, The use of lead citrate at high pH as an electron-opaque stain in electron microscopy. *J Cell Biol* **17**, 208-212 (1963).

10.	Q. Zhang *et al.*, *Schizosaccharomyces pombe* cells deficient in triacylglycerols synthesis undergo apoptosis upon entry into the stationary phase. *J Biol Chem* **278**, 47145-47155 (2003).
